# Association of age at menarche and menopause, reproductive lifespan, and stroke among Chinese women: results from a national cohort study

**DOI:** 10.7189/jogh.15.04154

**Published:** 2025-05-16

**Authors:** Lu Chen, Xisuo Lu, Xin Wang, Zhen Hu, Linfeng Zhang, Congyi Zheng, Zuo Chen, Xue Cao, Yixin Tian, Zengwu Wang

**Affiliations:** 1 Department of Nephrology, First Medical Center of Chinese PLA General Hospital, State Key Laboratory of Kidney Diseases, National Clinical Research Center for Kidney Diseases, Beijing Key Laboratory of Medical Devices and Integrated Traditional Chinese and Western Drug Development for Severe Kidney Diseases，Beijing Key Laboratory of Digital Intelligent TCM for the Preventionand Treatment of Pan-vascular Diseases, Key Disciplines of National Administration of Traditional Chinese Medicine(zyyzdxk-2023310), Beijing, China; 2 Department of Information, Beijing Friendship Hospital, Capital Medical University, Beijing, China; 3 Division of Prevention and Community Health, National Center for Cardiovascular Disease, National Clinical Research center of Cardiovascular Disease, State Key Laboratory of Cardiovascular Disease, Fuwai Hospital, Peking Union Medical College & Chinese Academy of Medical Sciences, Beijing, China

## Abstract

**Background:**

The association between age of menarche, age of menopause, reproductive lifespan and risk of stroke in Chinese women remains unclear and requires further clarification.

**Methods:**

A stratified multi-stage random sampling method was used to select participants at baseline in 2012–2015. The participants’ basic information was collected through a standardised questionnaire by professional investigator and examined by trained medical personnel. Follow-up was conducted in 2018–2020 to collect the stroke events. The Cox proportional hazards models were used to evaluate hazard ratios between reproductive factors and stroke risk.

**Results:**

Overall, 11 256 women (5155 non-menopausal women and 6101 menopausal women) were included (mean (x̄); standard deviation (SD) age = 55.2; 12.9 years). The risk was highest in women with menarche at age ≥17 years (hazard ratio (HR) = 1.290; 95% confidence interval (CI) = 0.959–1.733) and with reproductive lifespan ≤28 years (HR = 1.643; 95% CI = 1.041–2.595). Age at menarche was positively associated with risk of stroke (HR = 1.086; 95% CI = 1.006–1.172). There was a negative association between age at menopause and stroke risk in women with two live births (HR = 0.897; 95% CI = 0.834–0.964). Reproductive lifespan was negatively associated with risk of stroke (HR = 0.963; 95% CI = 0.946–1.027). Subgroup analysis also showed that association between age at menarche, reproductive lifespan and stroke events.

**Conclusions:**

Chinese women with late age at menarche and shorter reproductive lifespan have higher risk of stroke according to a large prospective study.

Stroke is the third most common cause of death in women and men, after heart disease and cancer, along with being one of the principal causes of disability [1]. Studies demonstrate that there are some important differences between the sexes that younger females are better protected from stroke than men [2]. This difference reverses as age advances in females and menopause ensues, resulting in higher incidence of stroke in females [2]. In addition, women report nonconventional symptoms of stroke more frequently than men which leads to delays in seeking medical attention, diagnosis, and accessing acute stroke services. Therefore, increasing awareness of the specific risk factors and warning signs of stroke that are unique to women may help reduce the total burden of cerebrovascular disease.

Menopause increases the stroke risk in females as corroborative from epidemiological study and animal studies [3,4]. This is thought to be because of the protective effects of ovarian hormones [5]. Hormonal factors, such as the decline in oestrogen and the relative increase in androgens in postmenopausal women, are believed to increase the risk and incidence of ischaemic stroke in this population [6]. This suggests that the increased risk of stroke in women may be related to reproductive factors, including age at menarche, age at menopause, and reproductive lifespan. Menarche is the beginning, and menopause is the end of a woman’s reproductive timeline, and the reproductive years, which is the interval between menarche and menopause, is a woman’s natural reproductive window. Due to exposure to different hormonal levels, early or late onset of these events may be associated with increased risk of many chronic health problems, in particular cardiovascular and cerebrovascular disease, such as stroke. Understanding reproductive lifespan and menstrual milestones is critical for stroke prevention. Reproductive chronology quantifies cumulative oestrogen exposure – each additional year reduces ischemic stroke risk by 5% through endothelial protection and lipid regulation. Notably, menarche ≥14 years elevates risk by 29% even premenopause, offering early detection windows [7]. Clarifying this effect could provide important information on new plausible paradigms to prevent stroke and ischemic vascular disease. However, the association between duration of reproductive factors and stroke risk has not been investigated thoroughly, which needs to be validated in large, long-term cohort studies.

Based on a national cohort study in China, we investigate the associations between age at menarche and menopause, reproductive lifespan, and the stroke risk and examine the relationship in subgroups amongst Chinese women.

## METHODS

### Study design and participants

**Figure 1:**
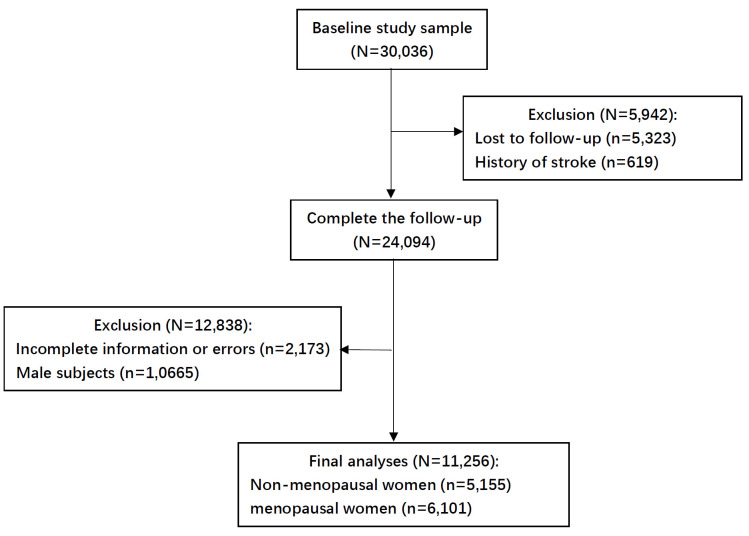
Flowchart of inclusion and exclusion of study participants.

From October 2012 to December 2015, our team carried out a representative large-scale cross-sectional survey of cardiovascular diseases (CVD) in China. A stratified multi-stage random sampling method was used to select about 500 000 participants aged ≥15 years from 262 districts and counties in 31 provinces in Chinese mainland. Demographic characteristics, lifestyle risk factors, drug therapy and female reproductive characteristics were collected. To obtain blood samples for biomarker analysis, a simple random sampling method was subsequently used to select participants aged ≥35 years from eligible areas. This resulted in a cohort of 30 036 participants with blood samples for investigation of fasting plasma glucose (FPG), total cholesterol (TC), triglycerides (TG), low-density lipoprotein cholesterol (LDL-C), and high-density lipoprotein cholesterol (HDL-C) levels. A total of 30 036 subjects who completed the baseline survey were followed up in 2018–2020. Of these, 5323 patients were lost to follow-up within five years, resulting in a total follow-up rate of 82.3%. After excluding 619 subjects with a history of stroke, 10 665 male participants, and 2173 postmenopausal women with missing or invalid data in key variables used in the analysis (*e.g*. age at menarche, age at menopause, contraceptive use, and breastfeeding history), a total of 11 256 women were included in the final analysis (**Figure 1**). Obtain written informed consent from each participant. The Ethics Committee of Fuwai Hospital (Beijing, China) approved the study

### Baseline measurement and data collection

Information on demographic characteristics, lifestyle risk factors, medical history and female reproductive characteristics was collected by professional investigator using standardised questionnaires. Height was measured using a standard right-angle meter and weight was measured using the Omron Body Fat Scale (V-body HBF-371, Omron, Japan) with participants wearing light clothing and barefoot. Blood pressure (BP) was measured in the right upper arm after the subject rested for at least five minutes in the sitting position using an Omron HBP-1300 professional portable sphygmomanometer (Omron, Kyoto, Japan). Laboratory tests were performed on venous blood samples taken after at least eight hours of overnight fasting. The levels of FPG, TC, TG, LDL-C and HDL-C were determined by the Central Core Laboratory (Beijing Adicam Clinical Laboratory Co., LTD., Beijing, China).

### Follow-up and outcome measures

During the follow-up phase, the occurrence of stroke events was tracked through interviewing participants or their agents, or through telephone or mail questionnaires, and medical records were further checked for reconfirmation. The participants' stroke events were initially recorded by local investigators, then the hospital records were reviewed by the Central Evaluation Committee of Fuwai Hospital (Beijing, China) to determine the final diagnosis. Stroke (ICD10 code I60–I61 and I63–I64) events are defined as an acute cerebrovascular disease caused by stenosis, occlusion or rupture of an artery in the brain due to various predisposing factors, resulting in an acute cerebral blood circulation disorder and limited or diffuse cerebral deficits, was divided into two types: ischaemic stroke and haemorrhagic stroke.

### Variable definition

At baseline, each participant was asked about their age at the onset of their first period and menopause, which were recorded as age at menarche and age at menopause. According to the World Health Organization, menopausal status was defined as the cessation of menstruation for at least 12 months. Reproductive lifespan was measured by subtracting the age at menarche from the age at menopause.

Body mass index (BMI) was calculated by dividing weight (kilogrammes (kg)) by height squared (square metres (m^2^)). Body mass index between 24.0 ~ 27.9 kg/m^2^ was defined as overweight, and BMI≥28.0 kg/m^2^ as obese [8]. Alcohol drinking was defined as consuming alcoholic beverages at least once a week in the past month. Smoking was defined as having smoked at least 20 packs of cigarettes in a lifetime and still smoking [9]. Diabetes was defined as FBG≥7.0 millimoles per litre (mmol/L) or a doctor’ s diagnosed of diabetes or taking hypoglycaemic drugs within two weeks [10]. Dyslipidaemia as TG≥2.26 mmol /L or TC≥6.22 mmol /L or HDL-C<1.04 mmol/L or LDL-C≥4.14 mmol/L or previous use of lipid-lowering drugs or previous diagnosis of dyslipidaemia according to the Guidelines for Dyslipidaemia in China [11]. Drug treatment was defined as taking any kind of anti-hypertensive, hypoglycaemic, or anti-hyperlipidaemia drugs. Comorbidity were any combination of two or more of three diseases: hypertension, diabetes and dyslipidaemia [12]. Parity was defined as the number of live births a woman gives birth to, whether or not the baby dies after delivery [13].

### Statistical analysis

Basic characteristics of participants were expressed as mean and standard deviation (SD) of the normally distributed data or as a proportion of the categorical data. The *x*^2^ test and variance analysis were used to compare variables among groups. Multivariable Cox proportional hazard model was used to evaluate hazard ratios (HRs) and 95% confidence intervals (CIs) for stroke events associated with age at menarche (classified as ≤13, 14, 15, 16, and ≥17 years) with 15 years as the reference group, age at menopause (classified as ≤44, 45-46, 47-48, 49-50, and ≥51 years) with 47–48 years as the reference group, and reproductive lifespan (classified as ≤28, 29–31, 32–34, 35–37, and ≥38 years) with 32–34 years as the reference group.

This study further investigated the associations between stroke and menarche by adjusting for age at recruitment (continuous), BMI (continuous), region (urban or rural), marital status (unmarried/widowed, married/cohabiting), education level (elementary or below, junior high school, high school or above), alcohol drinking (yes or no), smoking (yes or no), drug treatment (yes or no), comorbidity (yes or no), family history of stroke (yes or no), menstrual status(yes or no), age at menarche (continuous), reproductive lifespan (continuous), age at menopause (continuous), contraceptive use (yes or no), breastfeeding experience (yes or no) and parity (0–1, 2, ≥3). We also examined the stroke risk in subgroups of women defined by region, age at recruitment, marital status, BMI, education level, comorbidity, drug treatment, alcohol drinking status, and family history of stroke, use of contraceptives, parity. *R*, version 3.6.2 (R Foundation for Statistical Computing, Vienna, Austria) was used for analyses. The threshold of statistical significance was *P* < 0.05.

## RESULTS

The mean (x̄) (SD) age of participants was 55.2 (SD = 12.9) years and menopausal women was 63.4 (SD = 9.9) years. The mean age at menarche, age at menopause, reproductive lifespan and BMI and parity were 15.3 (SD = 1.8) years, 48.6 (SD = 3.6) years, 32.9 (SD = 3.9) years, 24.6 (SD = 3.6) kg/m^2^ and 2.4 (SD = 2.3), respectively. Those who smoked, drank, used contraceptives and had breastfeeding experience accounted for 2.5, 6.9, 9.2, and 95.4%, respectively, (**Table 1**, **Table 2**, **Table 3**).

**Table 1:** Characteristics of study participants by age at menarche

Characteristics*	Age at menarche						*P-*value
	**≤13**	**14**	**15**	**16**	**≥17**	**All**	
Number (%)	1860	1990	2064	1953	3389	11256	
Age at recruitment							
*Age, mean (SD)*	50.7 (12.4)	51.6 (12.2)	54.4 (13.0)	55.7 (1.7)	60.1 (12.0)	55.2 (12.9)	<0.05
*<50, n (%)*	1054	1059	921	723	731	4488	
*50–70, n (%)*	605	698	798	907	1887	4895	
*≥70, n (%)*	201	233	345	323	771	1873	
Baseline characteristics							
*Urban resident, n (%)*	1045	944	847	808	1248	4892	<0.05
*Have married, n (%)*	1682	1768	1799	1701	2793	9743	<0.05
BMI							
*BMI (kg/m^2^), mean (SD)*	24.9 (3.6)	24.6 (3.5)	24.6 (3.6)	24.7 (3.6)	24.5 (3.6)	24.6 (3.6)	<0.05
*<24 kg/m^2^, n (%)*	812	939	977	893	1448	5069	
*24-28 kg/m^2^, n (%)*	737	786	774	749	1164	4210	
*≥28 kg/m^2^, n (%)*	371	325	373	371	537	1977	
Education level, n (%)							<0.05
*Elementary or below*	738	930	1246	1218	2200	6332	
*Junior high school*	547	671	563	531	670	2982	
*High school or above*	633 ()	447 ()	313 ()	262 ()	287 ()	1942	
Alcohol drinking, n (%)	201 (10.8)	144 (7.2)	101 (4.9)	135 (6.9)	199 (5.8)	780 (6.9)	<0.05
Smoking, n (%)	43 (2.3)	43 (2.2)	37 (1.8)	64 (3.2)	91 (2.7)	278 (2.5)	0.011
Have comorbidity, n (%)	344	313	385	391	753	2186	<0.05
Have pharmacological treatment, n (%)	378	365	460	468	953	2624	<0.05
Ever use of contraceptives, n (%)	190 (10.2)	188 (9.4)	197 (9.5)	184 (9.4)	278 (8.2)	1037 (9.2)	0.405
Have breastfeeding experience, n (%)	1721 (92.5)	1907 (95.8)	1962 (95.1)	1877 (96.1)	3281 (96.8)	10748 (95.4)	<0.05
Parity							
*Parity, mean (SD)*	1.9 (1.2)	2.2 (3.3)	2.4 (1.5)	2.5 (2.6)	2.8 (2.2)	2.4 (2.3)	<0.05
*0–1, n (%)*	789	728	571	456	497	3041	
*2, n (%)*	650	716	747	763	1084	3960	
*≥3, n (%)*	421	546	746	739	1501	3948	
Have menopause, n (%)	713	820	1040	1098	2430	6101	<0.05
Family history of stroke, n (%)	112	96	108	113	213	642	<0.05
Follow-up event							
*Fatal and non-fatal stroke, n (%)*	26	26	27	44	91	214	<0.05

BMI – body mass index, SD – standard deviation

*Percentages were calculated based on women with complete information for that specific variable.

**Table 2:** Characteristics of study participants by age at menopause

Characteristics*	Age at menopause						*P-*value
	**≤44**	**45-46**	**47-48**	**49-50**	**≥51**	**All**	
Number (%)	666	717	1087	1974	1657	6101	
Age at recruitment							
*Age, mean (SD)*	61.5 (11.3)	62.5 (10.5)	62.6 (10.2)	64.55 (9.8)	63.7 (8.6)	63.4 (9.9)	<0.05
*<50, n (%)*	108	96	105	50	10	369	
*50–70, n (%)*	379	420	712	1294	1211	4016	
*≥70, n (%)*	179	201	270	630	436	1716	
Baseline characteristics							
*Urban resident, n (%)*	221	269	446	976	711	2623	<0.05
*Have married, n (%)*	524	564	878	1564	1362	4892	0.280
BMI							
*BMI (kg/m^2^), mean (SD)*	24.4 (3.6)	24.7 (3.5)	24.6 (3.6)	24.7 (3.7)	25.0 (3.7)	24.7 (3.7)	<0.05
*<24 kg/m^2^, n (%)*	307	308	491	895	698	2699	
*24–28 kg/m^2^, n (%)*	259	290	399	712	640	2300	
*≥28 kg/m^2^, n (%)*	100	119	197	367	319	1102	
Education level, n (%)							0.114
*Elementary or below*	483	508	739	1371	1137	4238	
*Junior high school*	117	124	224	354	296	1115	
*High school or above*	66	85	124	249	224	748	
Alcohol drinking, n (%)	53	40	59	96	88	336	0.071
Smoking, n (%)	35	30	32	67	44	208	<0.05
Have comorbidity, n (%)	163	174	287	532	522	1678	<0.05
Have pharmacological treatment, n (%)	212	221	343	689	610	2075	<0.05
Ever use of contraceptives, n (%)	44	60	103	124	137	468	0.014
Have breastfeeding experience, n (%)	641	681	1055	1896	1606	5879	0.339
Parity							
*Parity, mean (SD)*	2.9 (1.6)	3.0 (1.6)	2.9 (1.5)	3.0 (1.5)	2.9 (1.5)	2.9 (1.5)	0.243
*0–1, n (%)*	100	101	160	281	238	880	
*2, n (%)*	221	227	346	572	528	1894	
*≥3, n (%)*	345	389	581	1121	891	3327	
Age at menarche, mean (SD)	15.8 (2.1)	15.7 (1.9)	15.8 (1.9)	15.9 (2.0)	16.0 (1.9)	15.9 (1.9)	<0.05
Reproductive lifespan, mean (SD)	25.6 (2.8)	29.6 (2.0)	31.8 (2.0)	33.7 (2.1)	37.1 (2.7)	32.9 (4.2)	<0.05
Family history of stroke, n (%)	19	70	76	125	129	419	<0.05
Follow-up event							
*Fatal and non-fatal stroke, n (%)*	24	21	22	47	44	158	0.448

BMI – body mass index, SD – standard deviation

*Percentages were calculated based on women with complete information for that specific variable.

**Table 3:** Characteristics of study participants by reproductive lifespan

Characteristics*	Reproductive lifespan						*P-*value
	**≤28**	**29-31**	**32-34**	**35-37**	**≥38**	**All**	
Number (%)	827	1210	1826	1502	736	6101	
Age at recruitment							
*Age, mean (SD)*	63.0 (10.9)	63.3 (10.2)	63.7 (10.1)	63.5 (9.5)	64.2 (8.7)	63.5 (9.9)	<0.05
*<50, n (%)*	106	120	104	35	4	369	
*50–70, n (%)*	482	757	1198	1052	527	4016	
*≥70, n (%)*	239	333	524	415	205	1716	
Baseline characteristics							
*Urban resident, n (%)*	243	446	840	734	360	2623	<0.05
*Have married, n (%)*	639	951	1461	1220	621	4892	0.139
BMI							
*BMI (kg/m^2^), mean (SD)*	24.3 (3.4)	24.6 (3.6)	24.7 (3.8)	24.9 (3.6)	25.1 (3.6)	24.7 (3.6)	<0.05
*<24 kg/m^2^, n (%)*	383	554	812	644	306	2699	
*24–28 kg/m^2^, n (%)*	333	442	668	575	282	2300	
*≥28 kg/m^2^, n (%)*	111	214	346	283	148	1102	
Education level, n (%)							<0.05
*Elementary or below*	652	870	1280	960	476	4238	
*Junior high school*	117	223	348	288	139	1115	
*High school or above*	58	117	198	254	121	748	
Alcohol drinking, n (%)	58	68	94	66	50	336	<0.05
Smoking, n (%)	37	54	55	41	21	208	0.078
Have comorbidity, n (%)	195	292	501	444	246	1678	<0.05
Have pharmacological treatment, n (%)	261	380	622	522	290	2072	<0.05
Ever use of contraceptives, n (%)	57	87	157	119	48	468	0.778
Have breastfeeding experience, n (%)	800	1163	1777	1434	705	5879	0.070
Parity							
*Parity, mean (SD)*	3.0 (1.5)	3.0 (1.5)	3.0 (1.6)	2.8 (1.5)	2.8 (1.5)	2.9 (1.5)	<0.05
*0–1, n (%)*	110	130	249	269	122	880	
*2, n (%)*	249	398	566	439	242	1894	
*≥3, n (%)*	468	682	1011	794	372	3327	
Age at menarche, mean (SD)	16.9 (2.0)	16.8 (1.9)	16.1 (1.7)	15.1 (1.7)	14.6 (1.9)	15.9 (2.0)	<0.05
Age at menopause, mean (SD)	44.5 (0.82)	46.7 (1.71)	48.4 (1.6)	49.7 (1.2)	50.8 (0.5)	48.2 (2.4)	<0.05
Family history of stroke, n (%)	56	76	123	108	56	419	0.094
Follow-up event							
*Fatal and non-fatal stroke, n (%)*	30	25	44	43	16	158	<0.05

BMI – body mass index, SD – standard deviation

*Percentages were calculated based on women with complete information for that specific variable.

**Figure 2:**
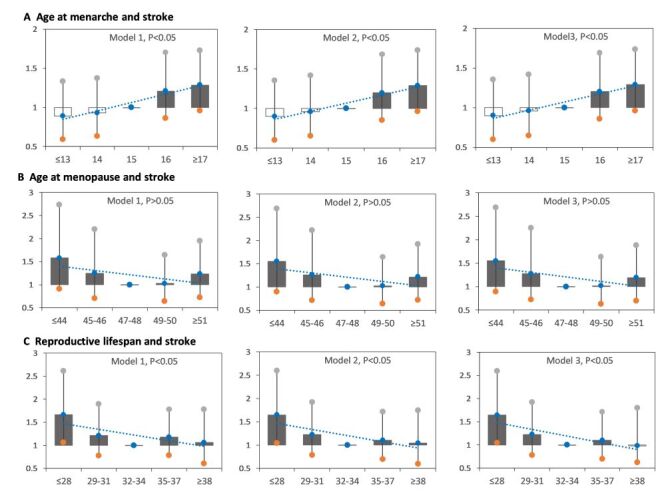
Hazard ratios (HRs) for stroke by age at menarche, age at menopause, and reproductive lifespan among women. Model A1, model B1, and model C1: adjusted for age at recruitment. Model A2, model B2, and model C2: model A1, model B1, and model C1 plus region, marital status, body mass index, education level, alcohol drinking, smoking, comorbidity, pharmacological treatment, family history of stroke. Model A3: model A2 plus menstrual status, contraceptive use status, and breastfeeding experience, parity; Model B3: model B2 plus age at menarche, reproductive lifespan, contraceptive use status, and breastfeeding experience, parity; Model C3: model C2 plus age at menarche, age at menopause, contraceptive use status, and breastfeeding experience, parity.

Age at menarche was positively associated with stroke events in Model 1, Model 2 and Model 3, while reproductive lifespan was negatively associated, age at menopause was not significantly. Model 3 showed that the HRs were 0.898 (95% CI = 0.596–1.353), 0.957 (95% CI = 0.647–1.414), 1.000 (reference), 1.202 (95% CI = 0.854–1.691), and 1.290 (95% CI = 0.959–1.733) for women with age at menarche ≤13, 14, 15 (reference), 16, and ≥17 years, respectively; 1.551 (95% CI = 0.893–2.690), 1.280 (95% CI = 0.720–2.250), 1.000 (reference), 1.016 (95% CI = 0.632–1.633), and 1.190 (95% CI = 0.699–1.883) for women with age at menopause ≤44, 45–46, 47–48 (reference), 49–50, and ≥51 years; and 1.643 (95% CI = 1.041–2.595), 1.225 (95% CI = 0.780–1.920), 1.000 (reference), 1.099 (95% CI = 0.694–1.711), and 0.980 (95% CI = 0.619–1.796) for women with reproductive lifespan ≤28, 29–31, 32–34 (reference), 35–37, and ≥38. The risk was highest in women with menarche at age ≥17 years and with reproductive lifespan ≤28 years (**Figure 2**).

**Figure 3:**
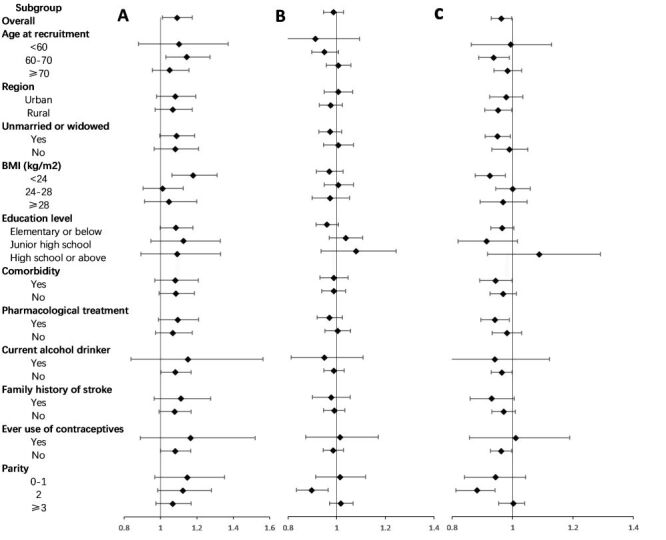
Adjusted hazard ratios (HRs) for stroke per year by age at menarche, age at menopause, and reproductive lifespan within various subgroups. **Panel A.** Adjusted HRs for age at menarche. **Panel B.** Adjusted HRs for age at menopause. **Panel C.** Adjusted HRs for reproductive lifespan. Model A were adjusted for age at recruitment, region, marital status, body mass index, education level, alcohol drinking, smoking, comorbidity, pharmacological treatment, family history of stroke, menstrual status, contraceptive use status, and breastfeeding experience, parity. Model B were adjusted for age at recruitment, region, marital status, body mass index, education level, alcohol drinking, smoking, comorbidity, pharmacological treatment, family history of stroke, age at menarche, reproductive lifespan, contraceptive use status, and breastfeeding experience, parity. Model C were adjusted for age at recruitment, region, marital status, body mass index, education level, alcohol drinking, smoking, comorbidity, pharmacological treatment, family history of stroke, age at menarche, age at menopause, contraceptive use status, and breastfeeding experience, parity.

After adjustment, the HR of stroke risk for age at menarche, age at menopause and reproductive lifespan was 1.086 (95% CI = 1.006–1.172), 0.986 (95% CI = 0.946–1.027) and 0.963 (95% CI = 0.929–0.998) per year. Subgroup analyses showed that age at menarche was positively associated with risk of stroke among women aged 60–70 years old, with low BMI, low education level, no alcohol consumption, and no previous contraceptive use. Reproductive lifespan was negatively associated with risk of stroke among women who were married, 60–70 years of age, low BMI, comorbidities, pharmacological treatment, not consume alcohol, not previous contraceptive use, and two live births. There also was a negative association between age at menopause and stroke risk in women with two live births, with an adjusted HR of 0.897 (95% CI = 0.834 ~ 0.964) per year (**Figure 3**).

## DISCUSSION

In this study, we first evaluated multiple subgroups using a nationally prospective sample to comprehensively investigate the associations between age at menarche and menopause, reproductive lifespan, and risk of stroke events in Chinese women. we found that older menarche and shorter reproductive years were related to an increased risk of stroke in Chinese women. The association was independent of age, BMI, alcohol consumption and contraceptive pill use. Improvement in stroke awareness in women of childbearing age is required.

Several studies have previously explored the association between age of menarche and stroke, but reported inconsistent results. The UK Million Women Study showed a significant U-shaped relationship between age at menarche and the outcomes stroke [14]. One cohort study reported that women with menarche age ≤13 years had a higher risk of ischemic stroke than those with ≤15 years, in which the participants were selected from a rural town in Japan [15]. In contrast, a study of 267 400 female textile workers in Shanghai found that early menarche was not associated with an increased risk of stroke mortality [16]. A case-control study reported that a higher age of menarche was associated with an increased risk of ischemic stroke, which was consistent with our study [17]. These discrepancies may arise from geographical and lifestyle differences, such as dietary habits and environmental exposures, as well as genetic backgrounds, which can influence health outcomes differently across regions [18,19].

In addition, the relationship between age at menopause and stroke risk has been contradictory in previous studies. Early menopause was positively associated with coronary heart disease and stroke in a longitudinal, multi-ethnic cohort study independent of traditional CVD risk factors [20], which might have certain limitations about extrapolation due to survival bias. Two meta-analysis indicated women with early menopausal age had a higher risk of stroke morbidity and mortality [21,22]. However, several studies found no convincing relationship between early menopausal age and CVD [16,23–27], which were consistent with our findings. Differences in population and study design, as well as the influence of confounding factors, may be the leading reasons for the inconsistent results.

Previous studies investigating the association between reproductive lifespan and stroke risk were inconsistent. Results from the US National Health and Nutrition Examination Survey showed that in women aged 60 and older, longer reproductive lifespan were associated with a lower risk of stroke [28]. A multi-centre case-control study in Taiwan showed that longer reproductive lifespan was associated with a lower incidence of stroke in postmenopausal women [17]. A meta-analysis found that women with a shorter reproductive lifespan was associated with a 31% increased risk of stroke [29]. One cross-sectional study from the USA showed that an annual increase in reproductive lifespan was associated with 3% reduction in the risk of overall CVD and stroke events [28]. For stroke mortality, a large prospective study from Japan showed a reduced risk for longer reproductive lifespan [30]. An analysis of the Nurses’ Health Study found that a reproductive lifespan of 30 years or less was independently associated with an increased risk of total stroke [31]. These associations between reproductive lifespan and the risk of stroke are consistent with our study. However, some studies have found no significant association [27,32–35] or even a U-shaped association [36]. Meanwhile, some studies have shown that there was a positive correlation between reproductive lifespan and the risk of stroke [37]. However, due to the lack of reproductive information, a representative prospective study is needed to verify the real association in the future.

Some mechanisms may explain the association between reproductive factors and risk of stroke. Studies have shown that late menarche is associated with low oestrogen levels [38,39]. Based on various experimental methods, oestrogen has been shown to affect haemorrhage volume, tissue survival, cerebral blood flow, and the immune response, all of which may affect the response to stroke and improve outcomes [32]. Timing of menarche and menopause were related, and women who experienced early menarche were at higher risk of early menopause [31]. A longer reproductive lifespan with reduced risk of stroke can be attributed to prolonged exposure to endogenous oestrogen [31]. In addition, changes in endogenous oestrogens may affect lipid levels, contributing to alteration in the lipoprotein profiles that may subsequently lead to atherosclerosis [33]. One potential mechanism to explain these changes in lipoprotein profile is that large high-density lipoprotein particles are converted to smaller particles by increased hepatic lipase, and oestrogen inhibits hepatic lipase [34]. Furthermore, recent findings indicate that oestrogen plays a crucial role in modulating fatty acid content and utilisation in the brain, which is vital for cerebrovascular health [40]. Oestrogen also enhances endothelial function by increasing nitric oxide production, thereby improving cerebral blood flow and reducing vascular tone [41]. Moreover, oestrogen's protective effects against atherosclerosis are mediated through the oestrogen receptor alpha/Sterol regulatory-element binding protein-1 signalling pathway, which regulates lipid metabolism and decreases LDL uptake [42]. Thus, the hypothesised mechanisms for these risks may be associated with shortened exposure time to beneficial endogenous estradiol and subsequent increases in higher-risk lipid profiles leading to atherosclerosis. These findings suggest that reproductive factors may play an important role in maintaining and improving cardiovascular health and could be used to assess populations with poor cardiovascular health for targeted interventions.

Our findings highlight women with late menarche and short reproductive lifespan as a high-risk group for stroke, suggesting targeted interventions. Clinically, this includes increased monitoring and lifestyle modifications. At the policy level, reproductive factors should be integrated into national screening programmes.

### Strengths and limitations

Our study has important advantages: prospective design with standardised methods and strict quality control measures; adjustments for potential risk factors for stroke and detailed subgroup analysis to improve the reliability of results. There are several limitations to consider. First, age at menarche and menopause is known to vary by ethnicity [35,36,43]. However, it is unclear whether ethnicity plays a role in this associations. Since the study participants were all Chinese women, which minimised the confounding effects of ethnic background, but might reduce generalisations to other races. Second, ages at menarche and menopause were self-reported, which increases the possibility of misclassification due to recall bias. However, previous studies have shown that the recall of menarche [44,45] and menopause age is relatively accurate [46,47]. Self-reported were a reasonably effective and repeatable method, which would be non-differential and tend to weaken the true strengths of the associations. Lastly, although our paper has comprehensively adjusted for potential confounding factors, the possibility of influence of other residual factors cannot be completely ruled out.

## CONCLUSIONS

Based on a national cohort study in Chinese women, we found that late age at menarche and shorter reproductive span were both significantly associated with increased risk of stroke. These associations remained consistent across subgroups. In addition, there was a negative association between age at menopause and stroke risk in women with two live births. Our findings have important public health implications for early detection and timely implementation of appropriate interventions in women at high risk of stroke. However, when applying these results to non-Chinese populations, caution is warranted due to potential cultural, lifestyle, and genetic differences.
